# Short-Term Plasticity in a Monosynaptic Reflex Pathway to Forearm Muscles after Continuous Robot-Assisted Passive Stepping

**DOI:** 10.3389/fnhum.2016.00368

**Published:** 2016-07-22

**Authors:** Tsuyoshi Nakajima, Kiyotaka Kamibayashi, Taku Kitamura, Tomoyoshi Komiyama, E. Paul Zehr, Kimitaka Nakazawa

**Affiliations:** ^1^Department of Integrative Physiology, Kyorin University School of MedicineMitaka, Japan; ^2^Faculty of Health and Sports Science, Doshisha UniversityKyoto, Japan; ^3^Motor Control Section, Department of Rehabilitation for the Movement Functions, Research Institute, National Rehabilitation Center for Persons with DisabilitiesTokorozawa, Japan; ^4^Graduate School of Engineering, Shibaura Institute of TechnologyTokyo, Japan; ^5^Division of Health and Sports Sciences, Faculty of Education, Chiba UniversityChiba, Japan; ^6^Rehabilitation Neuroscience Laboratory, University of VictoriaVictoria, BC, Canada; ^7^Graduate school of Arts and Sciences, University of TokyoTokyo, Japan

**Keywords:** spinal reflex, short-term plasticity, afferent feedback, passive stepping, humans

## Abstract

Both active and passive rhythmic limb movements reduce the amplitude of spinal cord Hoffmann (H-) reflexes in muscles of moving and distant limbs. This could have clinical utility in remote modulation of the pathologically hyperactive reflexes found in spasticity after stroke or spinal cord injury. However, such clinical translation is currently hampered by a lack of critical information regarding the minimum or effective duration of passive movement needed for modulating spinal cord excitability. We therefore investigated the H-reflex modulation in the *flexor carpi radialis* (FCR) muscle during and after various durations (5, 10, 15, and 30 min) of passive stepping in 11 neurologically normal subjects. Passive stepping was performed by a robotic gait trainer system (Lokomat^®^) while a single pulse of electrical stimulation to the median nerve elicited H-reflexes in the FCR. The amplitude of the FCR H-reflex was significantly suppressed during passive stepping. Although 30 min of passive stepping was sufficient to elicit a persistent H-reflex suppression that lasted up to 15 min, 5 min of passive stepping was not. The duration of H-reflex suppression correlated with that of the stepping. These findings suggest that the accumulation of stepping-related afferent feedback from the leg plays a role in generating short-term interlimb plasticity in the circuitry of the FCR H-reflex.

## Introduction

Part of the neuronal coordination between the fore- and hind-limbs observed in quadrupedal locomotion is preserved in humans (Dietz, 2002a; Zehr and Duysens, 2004; Sakamoto et al., 2007; Zehr et al., 2009; Meyns et al., 2014). This coordination is mediated by presumed locomotor generating systems and afferent feedback arising from limb movements and plays a functionally significant role in maintaining locomotor movements (Juvin et al., 2005; Nakajima et al., 2008a,b, 2011, 2013a,b, 2014; Zehr et al., 2009; Sasada et al., 2010). A useful method for assessing this coordination in the human spinal cord has been to measure the modulation of spinal reflex excitability during locomotion (Zehr et al., 2004; Zehr, 2005).

During rhythmic arm movement, Hoffmann (H-) reflex amplitude in stationary leg muscles is strongly suppressed (Frigon et al., 2004; Hundza and Zehr, 2009). Leg cycling also leads to H-reflex suppression in forearm muscles (Zehr et al., 2007; Nakajima et al., 2011, 2013b). Furthermore, a recent study we conducted revealed that robot-assisted passive stepping with a driven gait orthosis (DGO) induces forearm H-reflex suppression (Nakajima et al., 2011; Domingo et al., 2014). These interlimb interactions are partially regulated through presynaptic inhibition of group Ia afferent terminals in the H-reflex circuit (Frigon et al., 2004; Nakajima et al., 2013b). Thus, stepping-related afferent feedback from the legs may contribute to a decrease in transmission from Ia afferents to motoneurons (MNs) innervating forearm muscles.

The relationship between the extent of spasticity and hyperexcitable reflexes (Levin and Hui-Chan, 1993; Mezzarane et al., 2014) suggests that movement-related regulation of spinal reflexes could have therapeutic utility in the management of disorders of reflex control, such as spasticity after spinal cord injury and stroke (Zehr and Duysens, 2004; Hundza and Zehr, 2009; Nakajima et al., 2011; Mezzarane et al., 2014). In particular, for effective clinical translational application it is critical to determine both: (1) the minimum or effective duration for remote passive movement to elicit a long-lasting suppression of spinal reflexes; and (2) the underlying mechanisms and sources of any plasticity.

Brooke et al. (1992) demonstrated that the amplitude of the *soleus* (SOL) H-reflex was decreased during and after passive leg cycling in the supine position (Brooke et al., 1992; McIlroy et al., 1992; Misiaszek et al., 1995). The degree of the H-reflex suppression depended on the cadence, load, and limb position during leg cycling, and time after terminating leg cycling (Misiaszek et al., 1995). Generally, this suppression persisted for only 4 s after the cessation of movement. In addition, Motl and Dishman (2003) showed that acute bouts of active leg cycling for 30 min effectively attenuated the SOL H-reflex, but not the *flexor carpi radialis* (FCR) H-reflex. This previous work signifies that the H-reflex amplitude is strongly affected by the type of cycling movement (active or passive) and the limb position with respect to the limb where the H-reflex was recorded. Interestingly, Javan and Zehr (2008) demonstrated that 30 min of continuous rhythmic volitional arm movement could induce modulation of the SOL H-reflex amplitude for up to 20 min after the cessation of movement. However, it is unclear whether this effect was driven by descending voluntary commands or by afferent feedback from the movement itself. Hundza et al. (2012) showed that the acute effects of arm cycling had less to do with feedback and more to do with commands related to the frequency of the movement. Clearly both are involved but comparatively little is known about passive movement conditioning on reflex excitability in the arms particularly with regard to leg movements. The extent to which the short-term plasticity of reflex excitability within a limb muscle can be fully accounted for by the central drive or different sources of afferent feedback requires clarification.

It is yet to be determined whether persistent suppression of the H-reflex amplitudes in the arm muscles can be elicited by passive stepping-like movement of the leg in upright posture. In addition, the minimum stepping duration for inducing suppression of the H-reflex amplitude is a critical factor that remains uncertain. This information would be integral to effectively organize rehabilitation training for reducing exaggerated reflex excitability as found in spasticity.

Although the DGO was developed as a rehabilitation device for locomotion training after neurotrauma (Colombo et al., 2001; Dietz, 2002b; Kamibayashi et al., 2010), applying the DGO to healthy subjects is able to impose passive stepping in upright posture (Kamibayashi et al., 2010). Furthermore, DGO stepping with ground contact of the foot sole generates phasic loading in both the legs during the stance phase of stepping (Dietz, 2002a,b), and the afferent feedback from the load receptors plays a key role in driving locomotor systems in the spinal cord (Harkema et al., 1997; Van de Crommert et al., 1998; Dietz, 2002a,b; Nakajima et al., 2008a,b).

Therefore, the purpose of the present study was to determine the minimum effective duration of continuous passive leg stepping using DGO to induce long-lasting suppression of the forearm H-reflex amplitude. We hypothesized that long-lasting suppression of H-reflex amplitudes in FCR occurs after the termination of robot-assisted passive stepping and that the induced aftereffect is related to the duration of the stepping period.

## Materials and Methods

### Subjects

Eleven men (aged 22–32 years) with normal neurological functions provided informed written consent before participation in the study. The protocol of this project was approved by the local ethics committee of the National Rehabilitation Center for Persons with Disabilities and was conducted in accordance with the guidelines of the Declaration of Helsinki (1964).

### General Procedure

**Figure 1:**
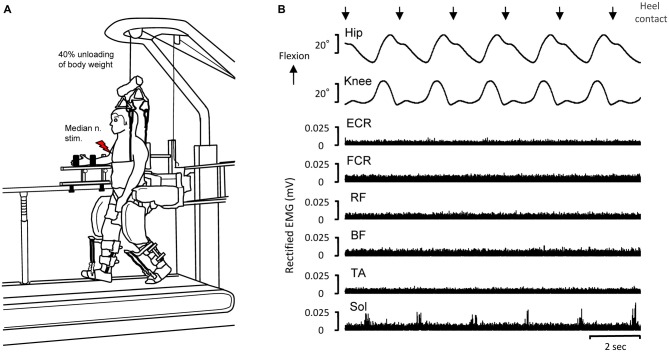
**Experimental set-up of driven gait orthosis (DGO) passive stepping (A) and typical recordings of joint angles and electromyographic (EMG) activity in the *flexor carpi radialis* (FCR), *extensor carpi radialis* (ECR), *biceps femoris* (BF), *rectus femoris* (RF), *tibialis anterior* (TA) and *soleus***(SOL)** muscles obtained from a single subject during DGO stepping (B).** EMG data are full-wave rectified. Downward arrows indicate the timing of heel contact during passive DGO stepping. Red lightning bolt: median nerve stimulation (test stimulation for evoking the FCR H-reflex).

The Lokomat DGO system (Hocoma AG, Vokeltswil) was used to produce “passive stepping” (defined as stepping movements driven by the DGO while relaxing the leg muscles). In this DGO stepping, there may be some low-level, involuntary muscle activation (see Figure 1B; cf. Nakajima et al., 2011). The procedure and methodology are similar to that described in our previous studies involving passive leg stepping (Kamibayashi et al., 2010; Nakajima et al., 2011). Briefly, this system consists of a treadmill, a body weight unloading system, and two robotic actuators that are attached to the subject’s legs (Nakajima et al., 2011). The Lokomat^®^ is fully programmable, including the control of the knee and hip kinematic trajectories during stepping with body weight loading (Colombo et al., 2001; Nakajima et al., 2011).

During stepping, the right forearm, wrist, and hand were fixed to a handmade rigid platform to minimize any unwanted movement of the arm (Figure 1A). All trials were performed with the FCR muscle quiescent. During passive stepping (2.0 km/h), the subjects were instructed to completely relax and allow the lower limb movements to be imposed by the DGO. Dorsiflexion of the ankle joint during the stepping condition was achieved by passive foot lifters (spring-assisted elastic straps) to prevent foot drop during the swing phase of the gait cycle (Figure 1A; Kamibayashi et al., 2010; Nakajima et al., 2011). After passive stepping, subjects stood stationary on the treadmill with the Lokomat system for FCR H-reflexes measurement. Forty percent unloading of body weight in all stepping and standing trials was accomplished by suspending the body with a counterweight harness connected to an overhead crane (Figure 1A; Nakajima et al., 2011).

### Experiments

To explore the long-lasting effects of passive stepping on forearm H-reflex amplitudes, the subjects participated in two experiments using the Lokomat system. The first was an investigation of the long-lasting suppression of FCR H-reflex amplitude after 30 min of passive stepping (Experiment 1, *n* = 10). The second assessed the effects of stepping duration (5, 10, and 15 min stepping periods) on FCR H-reflex suppression (Experiment 2, *n* = 9). In each experiment, passive stepping and static standing trials were performed. Experiments 1 and 2 were conducted on separate days. In Experiment 2, three training sessions of 5, 10, or 15 min stepping periods were conducted on the same day. These training sessions were conducted in a randomized order with an intersession interval of approximately 30–40 min to allow H-reflex amplitude to return to the control values.

Additional control experiments were conducted using the Experiment 1 protocol. In the first experiment, H-reflex amplitude was measured after standing on the treadmill for 30 min without passive stepping (*n* = 5). The second measured maximum M-waves (M_max_) after 30 min of passive stepping (*n* = 5). The two control experiments were conducted on separate testing days.

### FCR H-Reflexes

FCR H-reflexes in the right arm were elicited by stimulation (rectangular pulse, 0.5 ms duration) of the median nerve with a constant current electrical stimulator (SEN-7023, Nihon Kohden, Japan; Zehr et al., 2007; Nakajima et al., 2011, 2013b). Bipolar stimulation electrodes were placed just proximal to the medial epicondyle of the humerus, around the cubital fossa (Figure 1A; Zehr et al., 2007; Nakajima et al., 2011, 2013b). Stimulation was provided during the early stance phase (200 ms after heel strike), defined by hip joint angles. These stimuli were randomly applied once every 2–3 step cycles (Nakajima et al., 2008b, 2011). H-reflexes were elicited every 5 min during stepping and every 3 min after stepping had ended.

H-reflex (10 traces) amplitudes were measured while standing (pre-stepping control, 16 min after the start of stepping and post-stepping at 0, 3, 6, 9, 12, 15, 18, 21, 24, 27, and 30 min) and stepping (at 0, 5, 10, 15, 20, 25, and 30 min). At the beginning of Experiments 1 and 2, recruitment curves of FCR H-reflex and M-wave were constructed to determine both the M_max_, and maximum H-reflex (H_max_) sizes during the static condition on the treadmill (Hundza et al., 2012). Stimulus strength was set to elicit an H-reflex size of approximately 80% of H_max_ on the ascending limb of the recruitment curve (de Ruiter et al., 2010). The consistency of the test stimulation was confirmed and adjusted by observing the shape and peak-to-peak amplitude of the small M-wave throughout the experiment (Nakajima et al., 2011).

### Electromyographic (EMG) Recording

Electromyographic (EMG) activity was recorded from the FCR, *extensor carpi radialis* (ECR), *rectus femoris* (RF), *biceps femoris* (BF), *tibialis anterior* (TA), and SOL on the right side. EMG signals were obtained with surface electrodes (SS-2096, Nihon Kohden, Tokyo, Japan) placed over the muscle belly after decreasing impedance of skin to below 10 kΩ by light abrasion and cleaning with alcohol. All EMG signals were amplified (1000×) and band-pass filtered between 15 Hz and 3 kHz via a bioelectrical signal amplifier system (MEG-6108, Nihon Kohden, Tokyo, Japan; Nakajima et al., 2011). All data signals were converted to digital data with an analog-to-digital converter system (Micro 1401, CED Co. LTD., UK) and recorded on a hard disk of personal computer with a sample rate of 5 kHz using Spike 2^®^ software (CED Co. LTD., UK; Nakajima et al., 2011).

### Data Analysis and Statistics

Peak-to-peak amplitudes of M-waves and H-reflexes during stepping and standing were normalized to the control values of H-reflex amplitudes. These values were compared using the 1-way repeated measurement of analysis of variance (ANOVA). Multiple comparisons were performed using Dunnett’s *post hoc* test.

Furthemore, a power analysis (G * power, 3.1.9.2, Heinrich Heine University, Germany) to measure the effect size was performed on our data set (Faul et al., 2007). The effect size for 1-way repeated measures ANOVA was determined as a *f* index where a small effect was 0.1, medium effect was 0.25, and large effect was 0.40 (Cohen, 1988). This power analysis is useful for determining if any failure to observe significant differences were due to small sample size.

As with other physiological recovery curves (Devanne et al., 1997; Tazoe et al., 2007; Klimstra and Zehr, 2008), the data for the recovery process of the FCR H-reflex after the termination of the 5-, 10-, 15-, and 30-min passive stepping tasks were fitted to a three-parameter sigmoid function variation of the logistic equation using the Levenberg-Marquard nonlinear least-mean-square algorithm (Tazoe et al., 2007; Sigmaplot 10, Systat Software Inc., San Jose, CA, USA). This method was finally adopted after confirming that the sigmoidal curve was the best of the five types of fit curves considered (i.e., linear regression, polynomial, power, logarithmic, and sigmoidal function). The sigmoidal fit produced significant coefficients for all four stepping durations (all *p* < 0.01). In contrast, significant correlation coefficients were not obtained when fitted with the other methods (*p* > 0.05). Sigmoidal curve fitting analyses were performed using Sigmaplot 10 software (Systat Software Inc., San Jose, CA, USA). Data are described as means ± standard errors (SEM) and significant differences were set at *p* < 0.05 in all cases. All statistical tests except for the curve fitting analyses were performed using SPSS software (Ver. 11; SPSS, Chicago, IL, USA). *F*-values and degrees of freedom were obtained after the Greenhouse-Geisser correction, where appropriate (cf. Nakajima et al., 2011).

## Results

### EMG Patterns and Joint Movements While DGO Passive Stepping

Figure 1B depicts representative recordings of EMG activity and joint angles while the 30 min of passive stepping (with 40% unloading of body weight) for a single subject. In this stepping, the hip and knee joint trajectories were fully controlled by the DGO system. Thus, the joint movements were highly reproducible. EMG activities of the FCR, ECR, BF, RF, and TA were essentially inactive during passive stepping while small but rhythmic EMG activity was visible in several stepping phases in the SOL (cf. Nakajima et al., 2011).

### Persisting Suppression of the FCR H-Reflex After 30 min of DGO Passive Stepping

**Figure 2:**
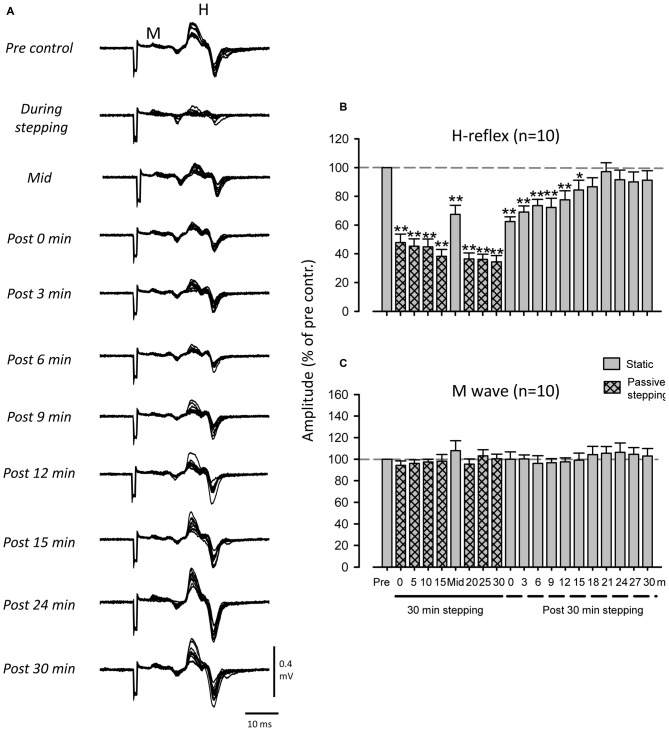
**Modulation of *FCR* H-reflex amplitude before, during, and after passive DGO stepping (40% unloading of body weight). (A)** Typical superimposed recordings of FCR H-reflex waveforms (10 sweeps) before, during, and after passive DGO stepping in a single subject. Grand means (±SEM) of the magnitude of the H-reflex **(B)** and M-wave **(C)** in the FCR obtained from 10 subjects before, during, and after 30 min of DGO passive stepping. Gray bars: during static conditions. Hatched bars: during DGO stepping conditions. **p < 0.01, *p < 0.05.

Figure 2A shows representative recordings of FCR H-reflexes during and after 30 min of passive stepping obtained from a single subject. The amplitude of the FCR H-reflex was strongly suppressed during passive stepping. Interestingly, H-reflex suppression was maintained for up to 15 min after the termination of passive stepping.

Figure 2B illustrates pooled data in the FCR H-reflex amplitudes obtained from 10 subjects. The mean amplitude of the FCR-reflex during DGO stepping was significantly lower than that during the pre-stepping control condition (Dunnett test; *p* < 0.001). After 30 min of stepping, reflex amplitudes were significantly suppressed for 15 min (Dunnett test; *p* < 0.05). The 1-way repeated ANOVA of H-reflex amplitudes described a main effect for time courses (*F*_(3.467,31.202)_ = 20.898, *f =* 4.250, *p* < 0.01). In contrast, we found no significant differences between the M-wave amplitudes in the control and test conditions (*F*_(3.974,35.765)_ = 0.988, *f* = 0.746, *p* > 0.05; Figure 2C).

### Effect of Stepping Duration on Prolonged Suppression of FCR H-Reflex

Experiment 2 was conducted to determine the duration of passive stepping required to induce prolonged suppression of H-reflex amplitudes. In Experiment 1, post-stepping suppression was maintained after 15 min of stepping (gray bar at “Mid” in Figure 2B). Therefore, in Experiment 2 we tested H-reflex modulation after 5, 10, and 15 min of stepping.

**Figure 3:**
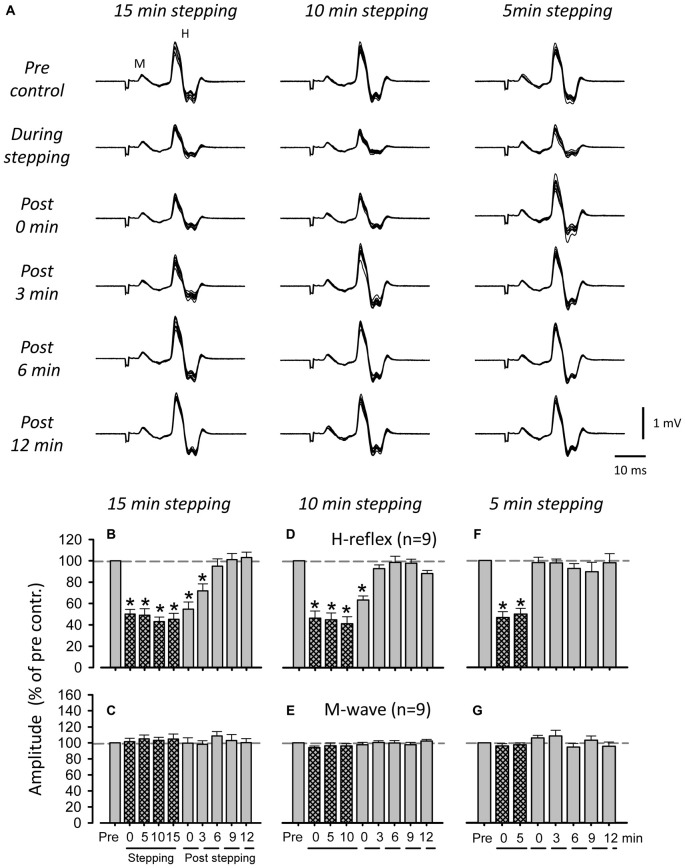
**Influence of passive stepping duration on FCR H-reflex amplitudes. (A)** Typical superimposed recordings (10 sweeps) of FCR H-reflex waveforms at 15 (left), 10 (center), and 5 min (right) of passive DGO stepping (40% unloading of body weight). Grand means (±SEM) of the magnitude of the H-reflex **(B,D,F)** and M-wave **(C,E,G)** in the FCR muscle obtained from nine subjects at 15 (left), 10 (center), and 5 min (right) of passive stepping (40% unloading of body weight). For each condition, FCR H-reflex waveforms were recorded every 5 min during stepping and every 3 min after stepping. *p < 0.01.

Figure 3A shows representative recordings of FCR H-reflexes during and after 5, 10, and 15 min of DGO passive stepping obtained from a single subject. Although suppression of H-reflex amplitudes after 10 and 15 min of passive stepping was observed (10 min: 0 min after stepping, 15 min: ≤ 3 min), it was not induced after 5 min of stepping. In Figures 3B–G, grouped data are shown for H-reflex and M-wave amplitudes evoked during and after 5, 10, and 15 min of stepping. In the 5 min stepping condition, there were no significant differences in H-reflex amplitude during any of the (0, 3, 6, 9, or 12 min) post-stepping periods [H-reflex: 1-way ANOVA; *F*_(7,18.439)_ = 17.648, *f* = 1.332, *p* < 0.001, Dunnett test; *p* > 0.05 (Pre vs. 0, 3, 6, 9 and 12 min after 5 min stepping), M-wave: 1-way ANOVA; *F*_(7,25.628)_ = 1.385, *f* = 0.416, *p* = 0.269] (Figures 3F,G). Significant suppression of the H-reflex amplitude after 10 min of stepping was only found immediately after the cessation of stepping (Figures 3D,E) [H-reflex: 1-way ANOVA; *F*_(8,21.926)_ = 38.489, *f* = 1.84, *p* < 0.001, Dunnett test; *p* < 0.001 (Pre vs. 0 min after 10 min stepping), M-wave: 1-way ANOVA; *F*_(8,17.178)_ = 1.022, *f* = 0.33, *p* = 0.386] whereas after 15 min of stepping, suppression was maintained for up to 3 min [H-reflex: 1-way ANOVA; *F*_(9,27.038)_ = 31.896, *p* < 0.001, *f* = 4.479, Dunnett test; *p* < 0.001 (Pre vs. 0 and 3 min after 15 min stepping), M-wave: 1-way ANOVA; *F*_(9,21)_ = 0.518, *f* = 0.20, *p* = 0.651] (Figures 3B,C). Thus, the duration of FCR H-reflex suppression after the termination of stepping was incremented according to stepping time.

**Figure 4:**
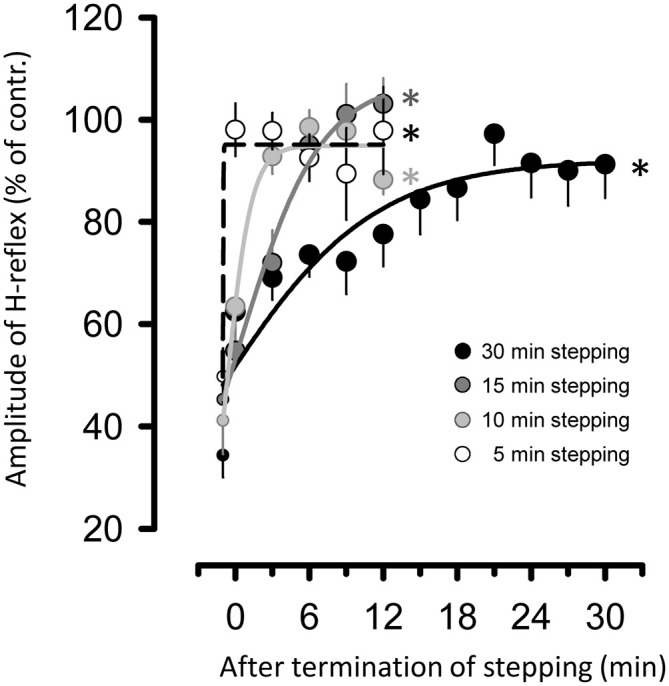
**Relationship between duration of passive DGO stepping [5- (opened circles), 10- (light gray circles), 15- (dark gray circles), and 30-min (filled circles) stepping tasks] and changes in amount of suppression of the H-reflex for up to 30 min after the termination of stepping.** To apply the curve-fitting analysis, data for suppression of H-reflex during stepping (i.e., last session of stepping in each task) were also used (small circles in each stepping task). The data were fitted with a three-parameter sigmoid function variation of the logistic equation using the Levenbrerg-Marquard nonlinear least-mean-square algorithm. All curve fits are significant. *p < 0.001.

Figure 4 illustrates the relationship between the duration of passive stepping and changes in H-reflex suppression for up to 30 min after the termination of stepping. The fit of all sigmoidal curves was statistically significant (5 min of stepping: *r* = 0.984, 10 min: *r* = 0.987, 15 min: *r* = 0.9952, and 30 min: *r* = 0.924, all *p* < 0.001). It is notable that the suppression of the H-reflex gradually tapering off with an increased duration of stepping. In fact, values of the slope parameter [*K* value, the Boltzman sigmoidal function (Devanne et al., 1997)] in curve fitting were well graded with the duration of passive stepping (5 min of stepping: 0.02, 10 min: 1.02, 15 min: 3.39, and 30 min: 6.27).

### Control Experiments

**Figure 5:**
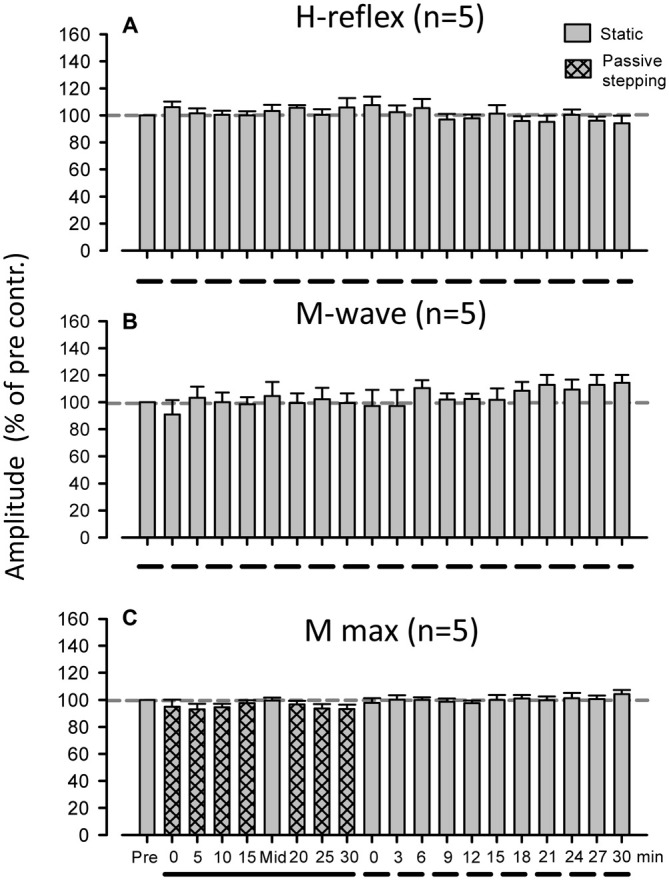
**Grand means (±SEM) of the magnitude of the H-reflex (A) and M-wave (B) in the *FCR* muscle obtained from five subjects during 60 min of standing. (C)** Grand means (±SEM) of the magnitude of the maximum M-wave in the FCR muscle obtained from five subjects before, during, and after 30 min of passive stepping. The additional control experiments were conducted using a protocol identical to that of Experiment 1. Filled bars and dashed lines indicate testing during static conditions and hatched bars and solid lines indicate during DGO stepping conditions.

During the entire 60-min experimental duration in the standing condition, FCR H-reflex amplitudes and M-waves were not significantly modulated (Figures 5A,B, *n* = 5; 1-way ANOVA: H-reflex: *F*_(2.104,8.418)_ = 1.296, *f* = 0.938, *p* > 0.05; M-wave: *F*_(2.758,11.03)_ = 0.212, *f* = 0.420, *p* > 0.05). In M_max_ experiment after 30 min passive stepping, also, a significant modulation of the M_max_ amplitude was not detected after 30 min of passive stepping (Figure 5C, *n* = 5; ANOVA: M_max_; *F*_(1.845,7.380)_ = 1.295, *f* = 0.468, *p* > 0.05).

## Discussion

In the present study, we demonstrated that passive stepping using a robotic device elicits prolonged suppression of monosynaptic reflex excitability in forearm muscles. In addition, the duration of FCR H-reflex suppression after the termination of stepping was correlated with the duration of the stepping period itself and decayed according to the slope parameter of a sigmoidal function. These findings suggest that continuous afferent feedback from passive stepping for more than 5 min can generate prolonged suppression of the forearm H-reflex amplitudes.

### Methodological Considerations

The amplitude of the M-wave in the FCR was used as an indication of the constancy of the afferent test volley (Zehr et al., 2007; Nakajima et al., 2011, 2013b). Similar M-wave amplitudes across all conditions indicated that the afferent volley induced by the various experimental conditions was relatively constant (Fukushima et al., 1982; Zehr, 2002; Pierrot-Deseilligny and Burke, 2005; Nakajima et al., 2011). Hence, there were no significant differences in M-wave amplitudes between any of the test durations (see Figure 2C). However, there is a possibility that the M_max_ amplitude changed over the course of the experiment (Crone et al., 1999). To examine this possibility, the M_max_ in the FCR was recorded in the control experiment and we confirmed that there were no significant differences in the M-wave amplitude during and after passive stepping (see Figure 5). Thus, the suppression of the H-reflex amplitude during and after passive stepping was not due to changes in the efficacy of the electrical stimulation delivered to the median nerve.

Reciprocal inhibitory effects arising from the forearm extensor muscles may also affect the amplitude of H-reflex in the FCR (Day et al., 1984; Nakashima et al., 1990; Nakajima et al., 2011, 2013b). However, the amplitude of the ECR EMG activity was minimal (see Figure 1) during and after stepping. Thus, the prolonged H-reflex suppression after passive stepping cannot be ascribed to a change in antagonistic ECR activities. Furthermore, H-reflex amplitude at rest in the Lokomat system did not significantly change over the same duration within the experimental session (see Figure 5A). Based on these results, it is unlikely that the number of electrical stimulations or experimental duration influenced persisting H-reflex suppression. Thus, we conclude that continuous passive stepping induced a prolonged suppression of the H-reflex amplitudes.

These previous studies showed that the H-reflex amplitude during or after cycling was strongly affected by the type of cycling (i.e., active or passive) and the limb from which the H-reflex was recorded (Brooke et al., 1992; McIlroy et al., 1992; Collins et al., 1993; Misiaszek et al., 1995; Motl and Dishman, 2003; Javan and Zehr, 2008). To our knowledge, ours is the first study to report prolonged suppression of the H-reflex in the arm muscle after robot-assist passive leg stepping while upright in humans. Taken together, methodological differences between the current study and previous reports may account for the prolonged suppression of the forearm H-reflex, such as posture (i.e., upright), the combination of limbs from which the H-reflex was recorded, and the limbs that passively moved. To date, the aftereffect of passive leg stepping while upright has not been previously reported.

### Possible Sources of the Prolonged Suppression of Forearm H-Reflex Amplitudes after Passive Stepping

Our finding that persistent H-reflex suppression in the forearm flexor muscles was induced by continuous passive stepping is in line those of previous studies (Javan and Zehr, 2008). However, one discrepancy between our study and the previous one is the possible contribution of voluntary drive to maintain leg movements during the locomotor task to elicit plastic changes in the excitability of the H-reflex pathway (Nakajima et al., 2011). In fact, EMG activities in the leg muscles during DGO stepping were extremely low, except for that in the SOL (see Figure 1). These small SOL EMG activities (0.5–2.0% of maximal voluntary contraction; MVC) were also confirmed in our previous works (Kamibayashi et al., 2009, 2010; Nakajima et al., 2011) even though the subjects were asked to completely relax. In these previous studies, we showed that EMG activity in the SOL might be a stretch-related muscle response, signifying that it could be involuntary in nature and/or activate a spinal pattern generator by the robot-assisted stepping task (Harkema et al., 1997; Nakajima et al., 2008a,b, 2011; Kamibayashi et al., 2009). These minimal EMG activations might be sufficient to drive these pattern-generating circuits (Harkema et al., 1997). During “normal” walking, however, it is acknowledged that the maximum EMG value in the SOL was ~80% of the MVC (Arsenault et al., 1986; Nishijima et al., 2010; Pery and Burnfield, 2010; Nakajima et al., 2011). Thus, it may be that the contribution of voluntary commands on lower leg muscles during passive DGO stepping was relatively small in our study. Considering all these observations, we believe that passive stepping-related afferent feedback from both legs, rather than voluntary commands from supraspinal regions, plays a key role in generating prolonged FCR H-reflex suppression.

Despite the foregoing, it is important to consider the contribution from supraspinal centers. Wagner et al. (2012) analyzed frequency content of electroencephalography (EEG) recordings during passive stepping and found both increased afferent feedback from the muscles to sensory cortex as well as increased activity in motor cortical “arm areas” during active and passive walking (cf., Müller-Putz et al., 2007; Jain et al., 2013; Obata et al., 2015; Jaeger et al., 2016). It is likely that similar cortical activities occurred in our study and may have been related to FCR H-reflex modulation. However, since our study was not designed to answer this question, it is difficult to substantiate whether these volleys from motor-related areas in the cortex drive to FCR and ECR MNs and neural circuits in the cervical cord. Thus, at this time we cannot give an explicit explanation for the presumed contribution of movement-related cortical activation on long-lasting H-reflex suppression after passive stepping. Further study is needed to elucidate this point.

Our findings could indicate that repetitive afferent feedback arising from leg muscles during passive stepping modulates the inhibitory neural networks that converge onto H-reflex pathways in the cervical cord and that this continues even after the cessation of passive stepping. During passive stepping, FCR H-reflex amplitudes were significantly attenuated (Nakajima et al., 2011), and it is well known that H-reflex suppression during rhythmic movement of the remote limb has been attributed to increased segmental group Ia presynaptic inhibition (Frigon et al., 2004; Nakajima et al., 2013b). Therefore, it is likely that inhibitory networks converging onto the forearm H-reflex arc were boosted during the passive stepping task in this experiment.

Based on these considerations, our findings suggest that passive stepping-induced afferent feedback modulates inhibitory systems and that this modulation plays an important role in the prolonged suppression of H-reflex amplitudes.

### Relation Between Prolonged Forearm H-Reflex Suppression and Stepping Duration

We found that the duration of the passive stepping strongly correlated to H-reflex suppression after the termination of stepping and decayed according to a slope parameter (*K* value) of a sigmoidal function. Long-lasting H-reflex suppression was not significantly seen after the 5 min stepping task in our condition. In other words, it is possible that 5 min of stepping-related afferent feedback was insufficient to induce long-lasting suppression of the monosynaptic reflex circuit in the cervical cord.

Although detailed mechanisms of the time dependency remain unclear, they may be explained by the “cumulative effect” of afferent feedback. In experiments with decerebrated cats, prolonged changes in MN excitability, or their sustained firing following continuous sensory input (e.g., group Ia afferents), were dependent on the duration of the stimulation (Crone et al., 1988; Hounsgaard et al., 1988). Interestingly, Crone et al. (1988) reported that maintained MN firing was correlated with the total amount of group Ia afferents (total duration of training stimulation and strength) in the stretch reflex pathway. This behavior may be a reasonable explanation for the long-lasting effect we observed after repetitive passive stepping. Javan and Zehr (2008) demonstrated the possibility of similar mechanisms in human inhibitory systems; an active rhythmic cycling movement induced persistent activation of presynaptic inhibitory interneurons impinging on group Ia terminals in the H-reflex circuit. However, further study is needed to explore the detailed mechanisms underlying this phenomenon.

It has been suggested that locomotor abilities may be regained after incomplete spinal cord injury and stroke with intense stepping training under the body-weight support (Van de Crommert et al., 1998; Dietz, 2002b; Nakajima et al., 2011). The suppressive effect of the stretch reflex circuit suggests therapeutic utility for passive stepping in the control of spasticity in remote muscles after spinal cord injury and stroke (Zehr and Duysens, 2004; Hundza and Zehr, 2009; Zehr et al., 2009; Nakajima et al., 2011; Mezzarane et al., 2014). However, our findings also indicate that passive stepping using a robotic device elicits long-lasting suppression of spinal reflex excitability in other segments of the spinal cord. Additionally, we determined the effective duration (10 min) of passive stepping for producing plasticity in this circuit.

A relationship has been reported between spasticity and exaggerated stretch reflexes (e.g., H-reflexes; Levin and Hui-Chan, 1993). Thus, our findings suggest that repetitive stepping-related afferent feedback could be a useful adjunct tool for spasticity management in remote limbs in affected patients. However, further studies are needed to better understand limitations to clinical utility.

## Author Contributions

Conceived and designed the experiments: TN, KK, ToK, EPZ, KN. Performed the experiments: TN, KK, ToK, TaK, KN. Analyzed the data: TN, KK, TaK. Contributed reagents/materials/analysis tools: TN, KK, KN. Wrote the article: TN, KK, ToK, EPZ, KN.

## Funding

This study (TN) was partially supported by the MEXT/JSPS KAKENHI (Nos. 19700461, 26560282).

## Conflict of Interest Statement

The authors declare that the research was conducted in the absence of any commercial or financial relationships that could be construed as a potential conflict of interest.
